# Anlotinib combined with TAS-102 as the third-line treatment for a patient with metastatic colon cancer: A case report

**DOI:** 10.3389/fonc.2022.978005

**Published:** 2022-11-30

**Authors:** Qizheng Li, Xia Zhang, Buqun Fan, Yudie Yang, Xiaonan Cui, Jie Zhang, Kaiteng Jiang, Chunxia Zhang, Bin Zhang

**Affiliations:** ^1^ Department of Oncology, The First Affiliated Hospital of Dalian Medical University, Dalian, China; ^2^ Department of Oncology, Dalian Fifth People’s Hospital, Dalian, China; ^3^ Queen Mary College, Nanchang University, Nanchang, China

**Keywords:** TAS-102, anlotinib, third-line, case report, metastatic colon cancer, mCRC

## Abstract

Chemotherapy combined with targeted therapy is a first-line and second-line treatment for metastatic colorectal cancer(mCRC), which has brought survival benefits to mCRC patients, however, disease progression is inevitable. More than 60% of patients still needed third-line treatment after the progress of second-line treatment. After the failure of second-line chemotherapy, treatment compliance and the physical tolerance of patients both decrease. Therefore, choosing an appropriate third-line treatment regimen is key to prolonging survival and improving quality of life. As a novel cytotoxic antitumor drug, trifluridine/tipiracil (TAS-102) is composed of trifluridine (FTD) and tipiracil hydrochloride (TPI). FTD can directly bind to the DNA of cancer cells to cause DNA dysfunction, thereby exerting antitumor effects. TPI can inhibit the degradation of FTD, thereby increasing its cytotoxicity. The few side effects of TAS-102 has become an important reason why clinicians present it as a treatment option to the patient for consideration, clinical trial data for progression free survival are lacking. The exploration of third-line treatment regimens with drug combinations has attracted much attention. This article reports a case of metastatic colon cancer (RAS/BRAF wild type, pMMR/Non-MSI-H), after failure of first-line and second-line therapies, the patient was eventually treated with anlotinib combined with TAS-102 as the third-line treatment. The treatment has shown good efficacy, with a long PFS benefit for more than 20 months and mild adverse reactions. This case reports demonstrates that anlotinib combined with TAS-102 is a promising third-line treatment regimen for refractory mCRC, and provides proof-of-concept for the clinical exploration of optimal third-line combination treatment regimens.

## Introduction

According to GLOBOCAN data, in 2020, the global incidence of colorectal cancer (CRC) ranked third among all malignant tumours, and the mortality rate ranked second (1). In China, the incidence of CRC ranks third among malignant tumours, and the mortality rate ranks fifth among malignant tumours (2). Surgery and radiotherapy are the primary local treatments. However, due to the high recurrence rate and metastasis rate, systemic treatment is critical for prolonging patient survival. Immunotherapy can bring significant benefits to mCRC patients with microsatellite instability-high (MSI-H)/deficient mismatch repair (dMMR), but for patients with microsatellite stability (MSS)/microsatellite instability-low (MSI-L)/proficient mismatch repair (pMMR) who account for the majority of mCRC patients, chemotherapy-based therapy remains the mainstay of treatment. Although chemotherapy combined with targeted therapy provides survival benefits for patients with metastatic CRC (mCRC), disease progression is inevitable. An Italian retrospective study showed that among mCRC patients who progressed after second-line treatment, 63.3% still needed third-line treatment (3). Third-line treatment regimens are limited, resulting in a 5-year survival rate of only 11% (4). After the failure of second-line chemotherapy, treatment compliance and the physical tolerance of patients both decrease. Therefore, choosing an appropriate third-line treatment regimen is key to prolonging survival and improving quality of life. Currently, the third-line treatment drugs of mCRC recommended by guidelines include regorafenib, fruquintinib, and trifluridine/tipiracil (TAS-102). TAS-102 is a novel cytotoxic antineoplastic agent with few side effects which has emerged as an important reason for clinicians to consider it as a third-line treatment option for mCRC patients, but clinical trial data for progression free survival (PFS)are lacking. The exploration of third-line treatment regimens with drug combinations has attracted much attention. Anlotinib hydrochloride is a novel multitarget tyrosine kinase inhibitors (TKI). In China, Anlotinib has been approved for the standard treatment of several solid tumors, and also some small-sample clinical studies have shown its effectiveness in the third-line treatment of mCRC. This article reports a patient with mCRC who failed first-line and second-line chemotherapy combined with targeted therapy, and third-line treatment with anlotinib combined with TAS-102 achieved good efficacy, thus providing a reference for clinical work.

## Case report

### Clinical data and initial treatment

**Figure 1:**
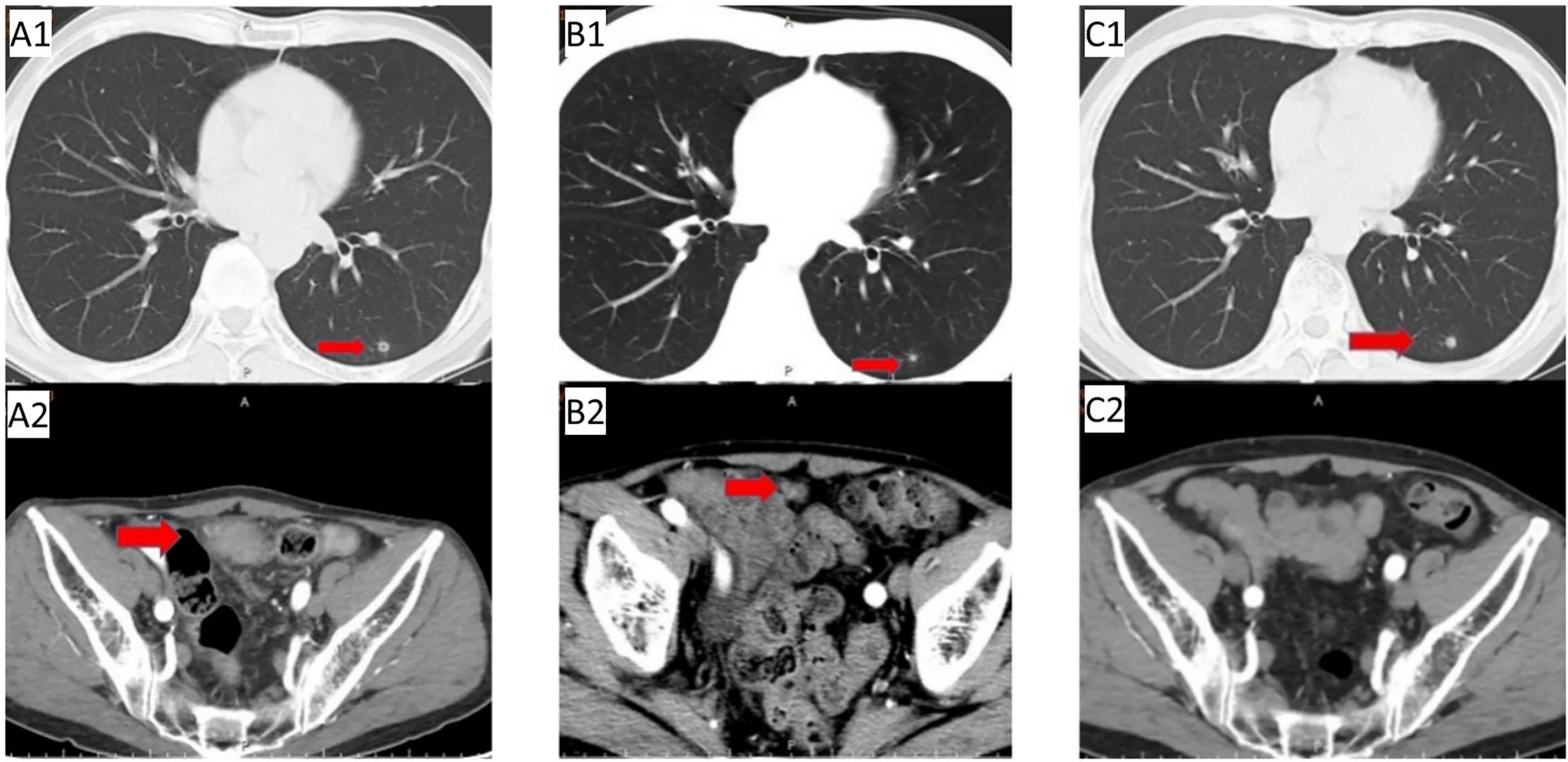
First-line treatment: **(A1/A2)** Baseline CT before first-line treatment; **(B1/B2)** CT after 2 cycles showed left lung nodules similar to before, pelvic metastases were significantly reduced; **(C1/C2)** follow-up CT on May 29, 2019 showed slightly enlarged left lung nodules, no pelvic metastases were observed.

The patient, a 65-year-old male, presented to the hospital on 12 July 2018, without obvious causes of abdominal pain and distention. He had an ECOG score of 1, and no special past history or family history. Abdominal computed tomography (CT) on 13 July 2018 revealed tumours at the beginning of the caecum and ascending colon, slight dilation of the proximal small intestine, and multiple slightly larger lymph nodes in the surrounding area; the tumours were considered malignant. Colonoscopy (2018-07-15) revealed a caecal ulcer-type lesion in the ileocecal region, with a high possibility of malignancy. Regarding pathology, the ileocecal tissue biopsy showed inflammation with high-grade focal glandular intraepithelial neoplasia. On 20 July 2018, laparoscopic radical resection of right colon cancer was performed under general anaesthesia. Postoperative dissection of the specimen revealed a mass in the ileocecal region, approximately 5 cm in size, with complete obstruction of the intestinal lumen. Postoperative pathology indicated (ileocecal) ulcerative moderately differentiated tubular adenocarcinoma, with a size of 4*2.5 cm, penetrating the serosa, with no clear vascular and nerve invasion and no cancer involvement at the small intestinal resection margin, large intestine resection margin, and peripheral resection margin. There was no cancer in the appendix, indicating inflammatory changes. No metastatic cancer was found in the peri-intestinal lymph nodes (0/14). The immunohistochemistry results were as follows: epidermal growth factor receptor (EGFR) (partial +), Ki-67 (+ approximately 65%), MLH-1 (expression), MSH-2 (expression), MSH-6 (expression), PMS-2 (expression), and P53 (+90%). The postoperative diagnosis was postoperative stage II ileocaecal bowel cancer (pT4aN0M0), pMMR ECOG 1. Re-examination of chest CT during postoperative adjuvant chemotherapy (30 August 2018) (**Figure 1A1**) revealed a small nodule shadow in the left lower lung; the nodule had a cavity. Pulmonary nodules were followed up closely after surgery. Postoperative adjuvant treatment of bowel cancer was initiated on 31 August, 2018: XELOX chemotherapy for 1 cycle – oxaliplatin 200 mg d1 ivgtt; capecitabine 1500 mg bid d1-14 po q21d.

### First-line treatment

On 3 September 2018, abdominal CT re-examination(**Figure 1A2**) due to abdominal pain revealed the following: 1. abdominal postoperative changes; 2. lower abdominal mass (metastasis was considered); and 3. a small amount of pelvic effusion. There was no definitive diagnosis. Positron emission tomography (PET)-CT examination on 4 September 2018 showed pelvic soft tissue nodules with increased 2-[fluorine-18]fluoro-2-deoxy-D-glucose (FDG) metabolism (metastatic tumour was considered) and small nodules in the lower lobe of the left lung with no increase in FDG metabolism; the small nodules were new compared to those initially observed on 26 October 2016. The following diagnoses were provided: 1. stage IV recurrence of ileocecal bowel cancer after surgery (rT0N0M1); 2. pelvic metastasis; 3. pulmonary nodular metastasis?; and 4. ECOG 1. Gene detection was completed: KRAS (WT)/NRAS (WT)/BRAF (WT). On 26 September 2018, first-line treatment (cetuximab + capecitabine and oxaliplatin (XELOX)) for mCRC was initiated, and the efficacy was evaluated as PR after 2 cycles (**Figure 1B1/B2**). After 3 cycles of treatment, the patient refused oxaliplatin treatment due to thrombocytopenia and neurotoxicity (numbness in the hands and feet). After the 4th cycle, cetuximab + capecitabine chemotherapy was initiated. Abdominal CT re-examination (20 February 2019) revealed the following: 1. postoperative changes in the abdomen; 2. lower abdominal mass (metastasis was considered); and 3. small amount of pelvic effusion. Efficacy was evaluated as PR. Abdominal and chest CT were conducted regularly at follow up.

### Second-line treatment

**Figure 2:**
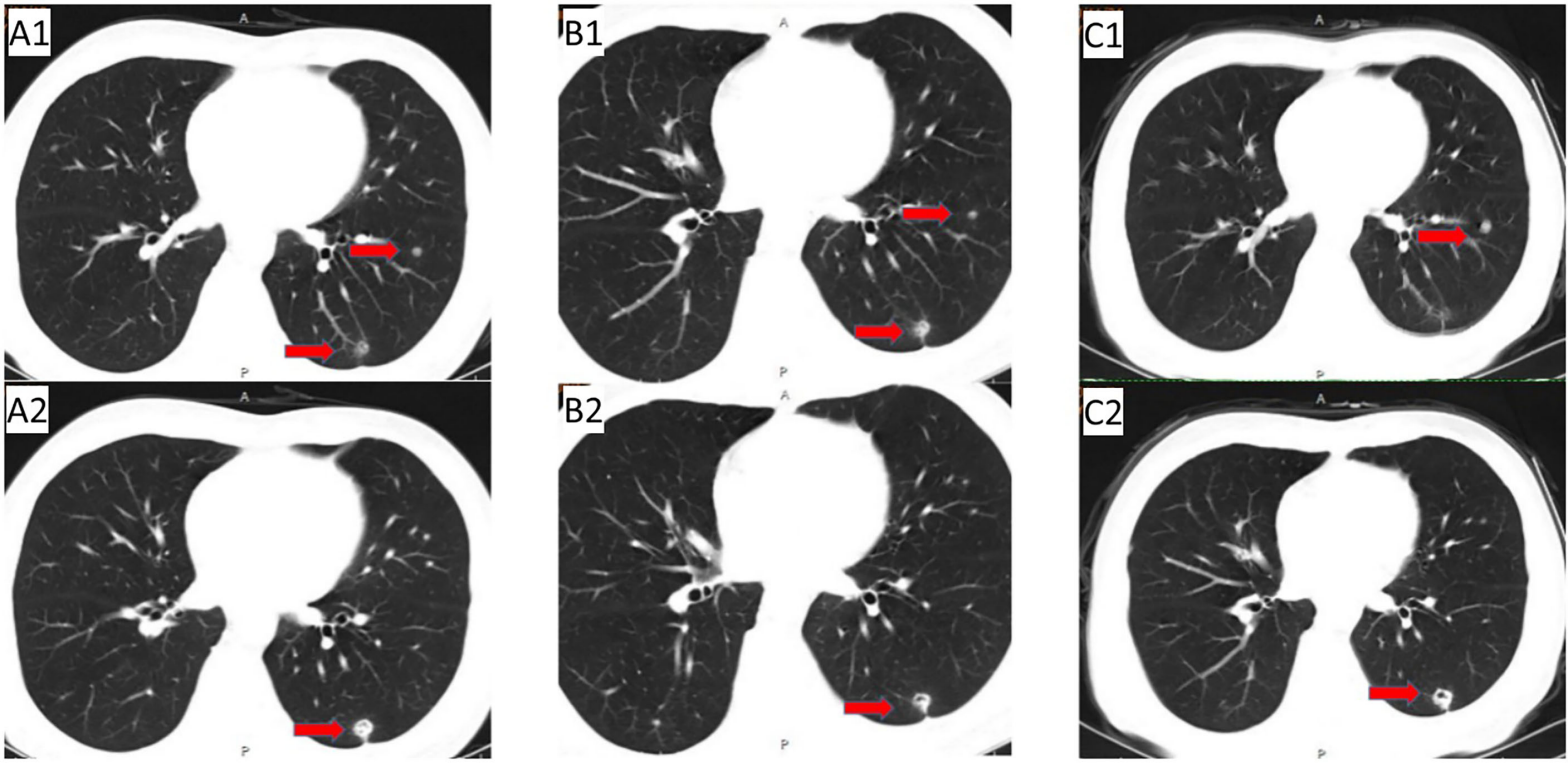
The period from the progression of lung metastasis to before the application of anlotinib + TAS-102 treatment: **(A1/A2)** Follow-up CT (2020.06.12): multiple nodules in bilateral lung; **(B1/B2)** CT (2020.08.12) after 1 cycle of second-line treatment; **(C1/C2)** initial application of cetuximab + TAS-102 in the third line, and discontinuation due to adverse reactions after only 2 cycles of cetuximab. On November 24, 2020, reexamination of CT showed that pulmonary nodules were increased and enlarged than before.

In May 2019, abdominal CT re-examination (**Figure 1C2**) showed no clear pelvic swelling, and chest CT (**Figure 1C1**) revealed small nodules in the left lower lung; the nodules were slightly larger than the nodules on previous imaging. The patient did not care about these changes. The patient felt that his physical condition was poor. He took Chinese medicine without consultation and was not admitted to the hospital. In June 2020, chest CT (**Figures 2A1/A2**)showed multiple nodules in both lungs, and metastasis was considered. On PET-CT, 1. the left lower lobe nodules were enlarged compared to the nodules on previous imaging, multiple new nodules developed in the remaining lungs, and FDG metabolism was increased in some lung regions. Metastatic tumours were considered. The pelvic nodules were significantly smaller than before, and the FDG metabolic activity was significantly lower than before; however, there was still residual tumour activity after treatment. Treatment with immune checkpoint inhibitors was recommended, but the patient refused the recommended treatment. Second-line chemotherapy with cetuximab + irinotecan for mCRC was initiated on 2 July 2020. After the first cycle, the patient refused to continue chemotherapy because of severe diarrhoea, and chest CT (**Figures 2B1/B2**) showed a reduction in the number of bilateral lung nodules.

### Third-line treatment

**Figure 3:**
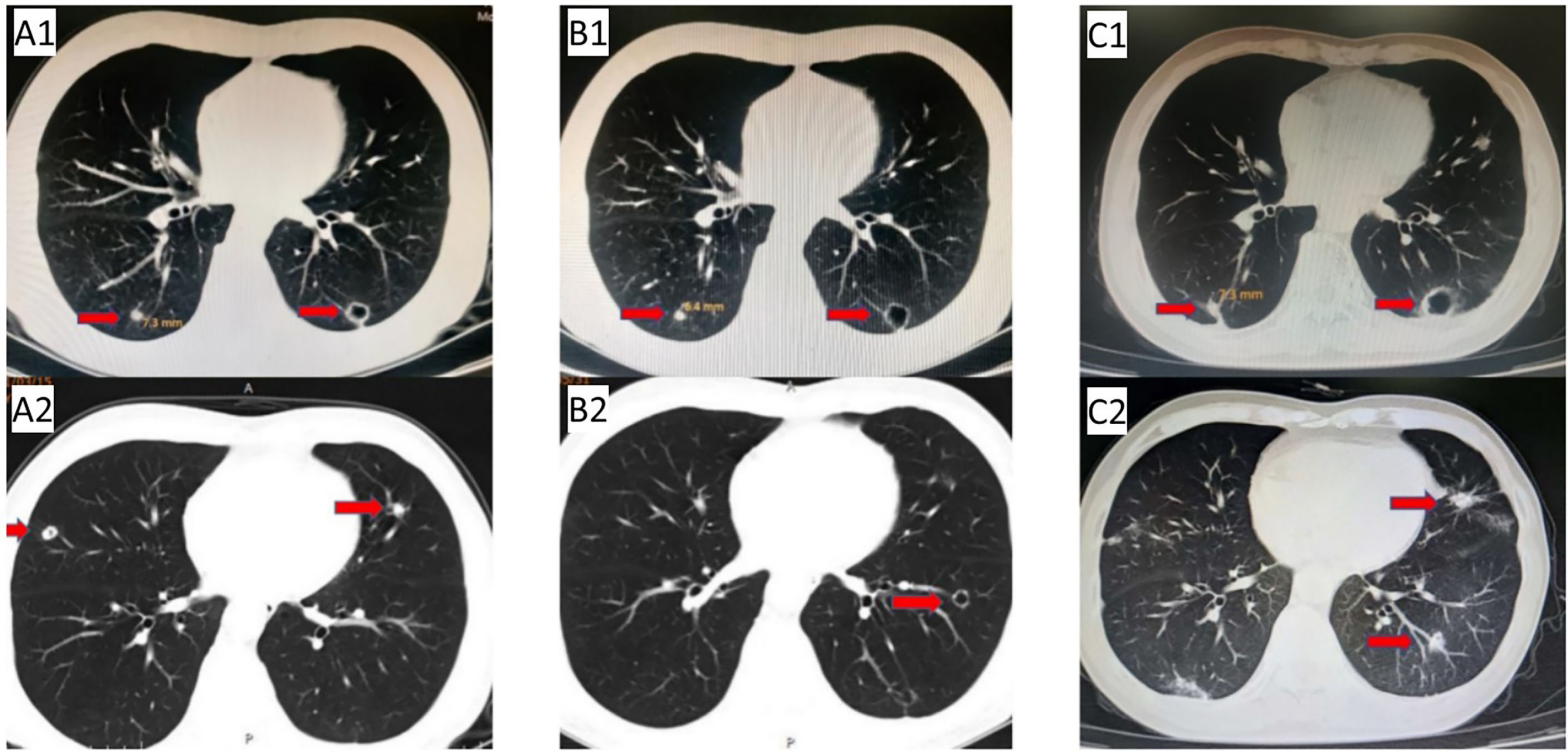
Anlotinib + TAS-102 treatment:**(A1/A2)** CT after 2 cycles (2021.03.15): the pulmonary nodules were larger than before (the right pulmonary nodules was 7.3 mm), and some cavities were formed; **(B1/B2)** reexamination CT (2021.05.31) showed that the right pulmonary nodules were smaller than before (6.4 mm); **(C1/C2)** last reexamination CT (2022.08.23): the right pulmonary nodules was 7.3 mm; multiple nodules in the left lung, and some cavities were formed.

**Figure 4:**
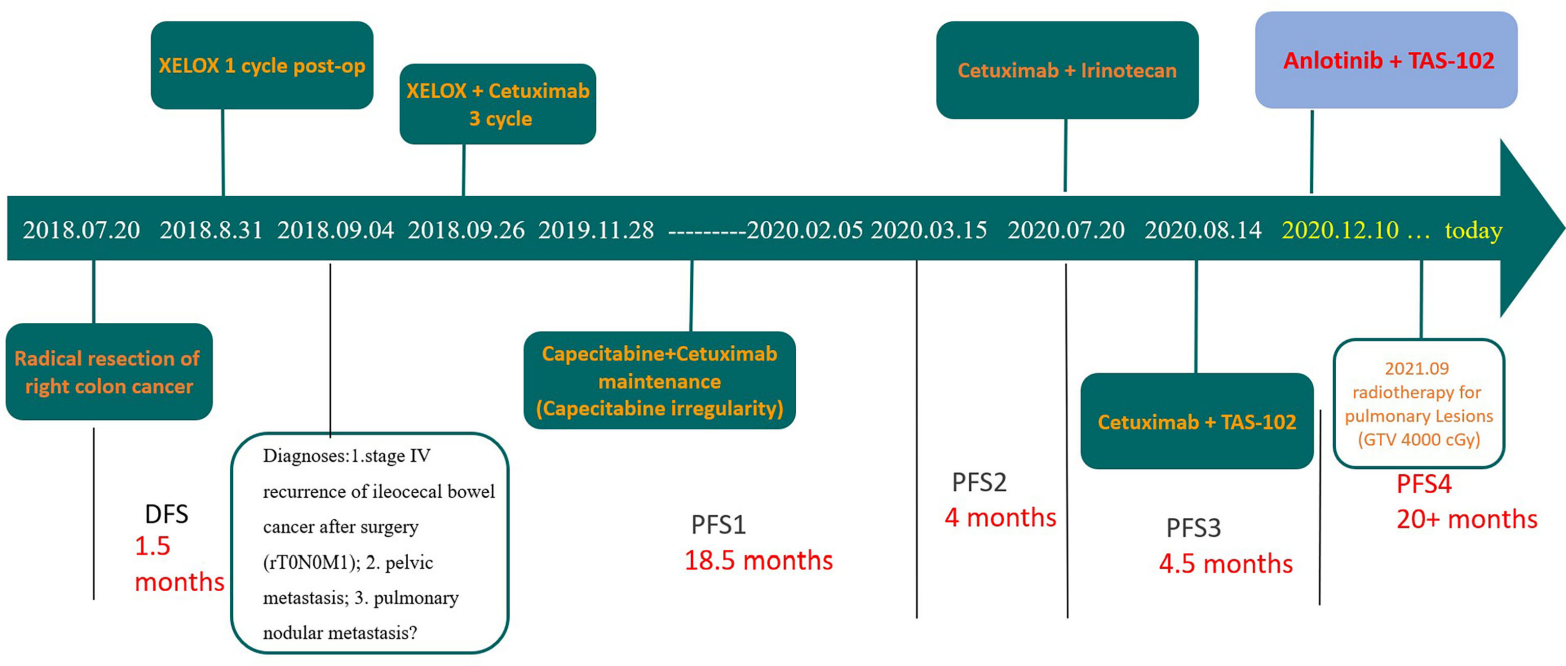
Diagnosis and treatment timeline.

Due to the serious side effects of previous chemotherapy, the patient rejected infusion chemotherapy and expected to choose oral drugs as much as possible. Therefore, TAS-102 was selected as the third-line treatment option for the patient. Considering that the benefits of third-line monotherapy of TAS-102 are limited, combined targeted therapy was suggested to improve treatment efficacy, which was accepted by the patient. Cetuximab + TAS-102 combined chemotherapy was initiated on 14 August 2020. After the first cycle, the patient had severe diarrhoea and grade IV granulocytopenia. Symptomatic treatment was administered to decrease diarrhoea and inflammation and elevate the WBC; cetuximab was discontinued after 2 weeks of application. Chest CT (24 November 2020) (**Figure 2C1/C2**) revealed that the number and size of pulmonary nodules increased. Considering that anlotinib is administered orally and some previous studies have shown that the adverse reactions caused by anlotinib in the treatment of mCRC are controllable, after informed consent of the patients, in December 2020, the third-line treatment regimen was adjusted to anlotinib + TAS-102 (TAS-102 40 mg d1-5, d8-12 bid po; q28d + anlotinib 10 mg d1-14 qd po; q21d). On 10 December 2020, the first cycle of anlotinib + TAS-102 treatment was initiated; the second cycle of anlotinib + TAS-102 treatment was initiated on 08 January 2021. Diarrhoea occasionally occurred; and granulocytopenia improved after treatment to elevate the WBC. The third cycle was not performed as scheduled due to the changes of precautionary measures over COVID-19 after the patient returned home, which prevented him from returning to the hospital. Abdominal CT re-examination (15 March 2021) showed no pelvic mass. On lung CT (15 March 2021) (**Figures 3A1/A2**), the bilateral lung nodules were larger than the nodule on previous imaging, and some cavities had formed. Anlotinib + TAS-102 treatment was continued on 17 March 2021, 28 April 2021, 9 June 2021, and 12 July 2021 for the 3rd through 6th cycles of treatment, respectively. Side effects of occasional diarrhoea and grade 1 granulocytopenia were observed. On 31 May 2021, abdominal CT showed no pelvic mass. Chest CT (**Figures 3B1/B2**) indicated that the pulmonary nodules were smaller than they were before and that some cavities had formed. SD was achieved by November 2021. In November 2021, the patient underwent multipoint radiotherapy for pulmonary lesions (GTV 4000 cGy, bilateral lung v5 40.13%, v20 10.34%). TAS-102 combined with anlotinib was continued after radiotherapy. The patient is still receiving treatment. The last re-examination (**Figures 3C1/C2**) was 23 August 2022. Efficacy was stable, and the PFS for Anlotinib + TAS-102 treatment was more than 20 months (**Figure 4**).

## Discussion

The choice of drugs for the third-line treatment of mCRCis critical. The KNOTE-177 study demonstrated a significant benefit from immunotherapy in patients with MSI-H/dMMR mCRC, however, this group of patients accounted for only 5% of all mCRC patients (5). For patients with MSS/MSI-L/pMMR who account for the majority of mCRC patients, chemotherapy-based treatment remains the mainstay of treatment, regorafenib, fruquintinib, and TAS-102 in the standard treatment regimen each have their advantages. The CORRECT study included patients with mCRC after failure of standard therapy in 16 countries from North America, Europe, Asia, and Australia, and showed that the overall survival (OS) for patients who received regorafenib target treatment was 1.4 months longer than that for patients in the placebo group (6.4 vs. 5.0 months; P=0.0052) (6). The CONCUR study confirmed that the efficacy of regorafenib in patients with advanced CRC in the Asia-Pacific region was better than that in Western patients; the median PFS of the regorafenib and placebo groups was 3.2 months and 1.7 months (p<0.001) and the median OS was 8.8 and 6.3 months (p<0.001) (7). The FRESCO study of the third-line treatment of mCRC with fruquintinib included 416 patients. The results showed that OS (primary endpoint) of the fruquintinib group and the placebo group was 9.30 months (95% confidence interval (CI), 8.18-10.45) and 6.57 months (95% CI, 5.88-8.11), respectively; the PFS (secondary endpoint) was 3.71 months (95% CI, 3.65-4.63) and 1.84 months (95% CI, 1.81-1.84), respectively. Regarding other secondary endpoints, the objective response rates (ORRs) were 4.7% and 0% (p=0.01), and the disease control rates (DCRs) were 62.2% and 12.3% (p<0.001) (8). However, for patients with good physical performance, the premature use of fruquintinib or regorafenib targeted therapy is not clinically desirable. Some doctors still try third-line chemotherapy, demonstrating that chemotherapy is still the main cornerstone of treatment in the minds of physicians, especially for non-MSI-H patients.

As a novel cytotoxic antitumor drug, TAS-102 is composed of trifluridine (FTD) and tipiracil hydrochloride (TPI). FTD can directly bind to the DNA of cancer cells to cause DNA dysfunction, thereby exerting antitumor effects. TPI can inhibit the degradation of FTD, thereby increasing its cytotoxicity (9, 10). TAS-102 has been shown to provide clinical benefit to patients with mCRC in several studies and has therefore been approved for third-line treatment of mCRC in several countries. RECOUSE, a global Phase 3 study involving 406 patients with refractory advanced colorectal cancer in China, Korea, and Thailand, demonstrated that compared with placebo, TAS-102 prolonged OS by 1.8 months (7.1 months vs. 5.3 months, p<0.001) and mPFS by 0.3 months (2.0 months vs. 1.7 months, p<0.001) in patients with drug-resistant refractory mCRC after standard second-line treatment, regardless of geographic origin, or KRAS status (11). The TERRA study on TAS-102 that included Asian patients with refractory mCRC who were resistant or intolerant to standard chemotherapies showed that the OS of the treatment group and the placebo group were 7.8 months and 7.1 months, respectively (p=0.035), and that the mPFS were 2.0 months and 1.8 months, respectively (p <0.001) (12).

The results from the above studies suggest that the benefits of third-line monotherapy are limited and that it is imperative to explore potential drug combination regimens for the third-line treatment of mCRC. Due to the excellent safety of TAS-102 monotherapy, many studies evaluated the efficacy and safety of TAS-102 in combination with other drugs for the treatment of refractory mCRC. The results of an open-label, single-arm, multicentre, phase 1/2 trial of TAS-102 plus bevacizumab initiated by Japanese investigators (C-TASK FORCE) showed that TAS-102 combined with bevacizumab for patients with mCRC who are resistant or intolerant to standard chemotherapy can achieve an mPFS of 5.6 months (95% CI, 3.4-7.6) (13). An open-label, randomized, phase 2 study in Denmark compared the efficacy of TAS-102 monotherapy and the combination of TAS-102 and bevacizumab in patients with refractory mCRC. The median OS obtained using the combination of TAS-102 and bevacizumab was 9.4 months (95% CI, 0.32-0.94), and the mPFS was 4.6 months (95% CI, 0.29-0.72). The mPFS of patients treated with TAS-102 monotherapy was 2.6 months (95% CI, 1.6–3.5) (14). The TAS-CC3 study is a prospective, nonrandomized, single-arm, multicentre, open-label phase II trial. In that study, for patients with mCRC, TAS-102 plus bevacizumab as a third-line treatment achieved a median PFS of 4.5 months (95% CI, 1.8-7.1) and median OS of 9.2 months (95% CI, 5.5-12.8) (15). In addition, in the APOLLON study, the median PFS and OS of patients with wild-type RAS mCRC treated with TAS plus panitumumab were 5.8 months (95% CI, 4.5-6.5) and 14.1 months (95% CI, 12.2-19.3), respectively (16). The results of these studies are encouraging, and thus, clinicians should further explore TAS-102 combination therapy for mCRC to extend time benefits for patients.

The patient in this case study had advanced colon cancer patient (wild-type Ras/BRAF gene; pMMR/Non-MSI-H). After second-line chemotherapy failed, the initial third-line chemotherapy regimen was cetuximab and TAS-102 because after indirectly comparing different drug regimens, the ORR/PFS/OS of cetuximab rechallenge therapy were all superior to those of other third-line therapy drugs, indicating that for RAS/BRAF WT mCRC patients, the application of cetuximab third-line rechallenge is also an optimized treatment strategy (17, 18). However, after treatment, the patient developed severe intolerable diarrhoea. As more and more evidence has proved the benefit of TAS-102 combined with bevacizumab in the third-line treatment of mCRC, and anlotinib also has some small-sample clinical studies in the third-line treatment of mCRC, and it is oral administration, anti-angiogenic therapy with anlotinib instead of bevacizumab seems to be more suitable for patients who are resistant to intravenous infusion. The regimen was adjusted to anlotinib in combination with TAS-102. Anlotinib hydrochloride is a novel multitarget tyrosine-kinase inhibitor (TKI) that can inhibit angiogenesis-related kinases, including VEGFR1/2/3, PDGFRα/β, FGFR1/2/3, c-Kit, Met, Ret, and Tie2. It can also inhibit tumour growth and metastasis by inhibiting a variety of tumour-associated kinase targets, such as EGFR, ALK, ABL, Aurora-A/B, DDR2, and EphB4 (19). Previous clinical studies have shown that anlotinib is effective for non-small-cell lung cancer (NSCLC), medullary thyroid carcinoma, and soft tissue sarcoma, with controllable adverse reactions (20). Anlotinib has been approved for the standard treatment of NSCLC, soft tissue sarcoma and small cell lung cancer in China. A multicentre, double-blind, placebo-controlled, randomized phase III trial (ALTER0703) enrolled 419 patients with refractory mCRC from 33 hospitals in China. In that study, the median PFS improved (4.1 months, 95% CI, 3.4 - 4.5). Subgroup analysis showed that in RAS/BRAF wild-type patients, anlotinib provided significant survival benefits. The authors of that study concluded that anlotinib significantly prolonged clinical benefits (PFS) for patients with refractory mCRC (21). In addition, a retrospective clinical study in China collected the clinical data of 105 mCRC patients from who failed at least two lines of chemotherapy, and the analysis showed that anlotinib was superior to chemotherapy as a third-line treatment of mCRC (PFS: 3.46 months vs 2.25 months,P< 0.001; OS: 9.22 months vs 6.95 months, P< 0.001), and similar to fuquinitinib or regorafenib (PFS: 3.46 months vs 3.33 months,P=0.347; OS: 9.22 months vs 9.38 months, P=0.499), and the related adverse reactions were tolerable (22).

From a number of completed clinical studies of TAS-102 or Anlotinib monotherapy in the treatment of mCRC, most of the adverse reactions of TAS-102 or anlotinib in the treatment of mCRC were tolerable and controllable. The most common adverse events associated with TAS-102 in RECOURSE were neutropenia (38%), leukopenia (21%) and neutropenic fever (4%) (11); in TERRA, the most common adverse events of grade 3 and above were neutropenia (33.2%), leukopenia (20.7%), and anaemia (17.7%). The adverse reactions can generally be controlled by reducing the dose, extending the interval between chemotherapy and administering relevant drugs for symptomatic management and are relatively manageable. The study also reported that TAS-102 was well tolerated in Asian patients with mCRC (12). The most common adverse reactions caused by anlotinib are fatigue, gastrointestinal toxicity, hypertension, proteinuria, rash, and hand-foot reactions. Most patients recover or improve after symptomatic treatment and drug dose reductions. The ALTER0703 study analysis concluded that for anlotinib in refractory mCRC, most common grade≥3 TRAEs were hypertension, increased γ-GT, and hand-foot skin reaction, the TRAEs were manageable,and the deterioration of QoL in anlotnib was as same as placebo for patients (21). For the patient in this case study, the side effects were only occasional diarrhoea and grade I granulocytopenia after adjusting the regimen to anlotinib + TAS-102.

To our knowledge, there are no reports on anlotinib combined with TAS-102 as a third-line treatment for patients with refractory mCRC. The treatment of the patient with advanced colon cancer in this study obtained the informed consent of the patient and family members. The patient was eventually treated with anlotinib combined with TAS-102 as the third-line treatment, rather than the standard third-line regimen of TAS-102 monotherapy, with informed consent. Medicine should be evidence-based and follow ethics, but individualized trials with patients’ informed consent may lead to better efficacy. The treatment has shown good efficacy, achieving a PFS benefit far beyond that of standard third-line therapy, with mild adverse reactions, and the patient is still receiving treatment. Radiation therapy has also been administered during treatment, resulting in stable disease control. This case reports demonstrates that anlotinib combined with TAS-102 is a promising third-line treatment regimen for refractory mCRC, and provides proof-of-concept for the clinical exploration of optimal third-line combination treatment regimens. As the previous TERRY study showed significant benefit of TAS-102 in Asian patients with mCRC, while subgroup analysis of ALTER0703 showed significant OS survival benefit of anlotinib in patients with RAS/BRAF wild-type, given that the patient reported in this case belonged to Asian non-MSI-H/pMMR and RAS/BRAF WT mCRC, whether this combination regimen of TAS-102+ anlotinib is more advantageous for this part of the population needs to be verified by further clinical studies.

## Data availability statement

The original contributions presented in the study are included in the article/supplementary material. Further inquiries can be directed to the corresponding authors.

## Ethics statement

Ethical review and approval was not required for the study on human participants in accordance with the local legislation and institutional requirements. The patients/participants provided their written informed consent to participate in this study. Written informed consent was obtained from the individual(s) for the publication of any potentially identifiable images or data included in this article.

## Author contributions

QL and XZ provided equal contribution to this work. BZ and XC contributed to the conception and design and provided administrative support. CZ provided necessary information. XZ and QL took charge of the collection and assembly of data, conducted the disease analysis, provided the summary. All authors contributed to the article and approved the submitted version.

## Funding

This work was supported by the Scientific research fund project of Education Department of Liaoning Province (LJKZ0861) and the Dalian Medical Science Research Project (No. 2011012) and the Natural Science Foundation of Liaoning province (Nos. 20180550077 and 20180550937).

## Conflict of interest

The authors declare that the research was conducted in the absence of any commercial or financial relationships that could be construed as a potential conflict of interest.

## Publisher’s note

All claims expressed in this article are solely those of the authors and do not necessarily represent those of their affiliated organizations, or those of the publisher, the editors and the reviewers. Any product that may be evaluated in this article, or claim that may be made by its manufacturer, is not guaranteed or endorsed by the publisher.
